# A new apameine genus and species from the southern Appalachian Mountains, USA (Lepidoptera, Noctuidae, Noctuinae)

**DOI:** 10.3897/zookeys.421.7727

**Published:** 2014-06-27

**Authors:** Eric L. Quinter, J. Bolling Sullivan

**Affiliations:** 1 P.O. Box 74, Willimantic, Connecticut, 06226, USA; 2 200 Craven Street, Beaufort, North Carolina, 28516, USA

**Keywords:** Apameini, *Arundinaria*, bamboo, barcode, biogeography, endophagy, evolution, Noctuoidea

## Abstract

*Cherokeea* **gen. n.** is proposed for a rarely collected apameine moth species from the southern Appalachian Mountains, C. *attakullakulla* **sp. n.** It is recorded from foothills and lower montane habitats of North Carolina and Georgia where hill cane, *Arundinaria appalachiana* Triplett, Weakley & L.G. Clark is found. Adults and their genitalia are figured and a mtDNA barcode sequence is given.

## Introduction

Most of the species formerly placed in the “*Oligia*” *semicana* (Walker) group were resolved by Troubridge and Lafontaine (2002) and placed in the new genus *Neoligia* Troubridge & Lafontaine. Omitted from that revision was a rarely-seen species from the southern Appalachian Mountains. First collected by John G. Franclemont in 1958 during his studies of the fauna at Highlands Biological Station in Macon County, North Carolina, the species remained uncollected until faunal studies began in the Great Smoky Mountain National Park in the late 1990’s. While conducting various lepidopteran surveys in Swain County, the species was rediscovered at numerous localities. Although it superficially resembles *Neoligia semicana* (Walker), an uncommonly collected species of the northeastern United States, genitalic dissections and DNA sequences at the CO1 locus reveal it is distinct at the generic level.

This paper describes the second of a number of new genera of apameine moths that are highly restricted to the limited occurrences of their known or presumed host plants, *Arundinaria* Michx. (Poaceae: Bambusoideae) that occur in the southeastern United States. The first new genus, *Protapamea* Quinter (Quinter 2009), and several additional new genera (Quinter, in prep.) share a suite of characters unique among the Nearctic and western Palearctic Apameini, suggesting an ancient lineage involving a common ancestor. With no obvious close relatives known in these regions, it seems likely that the closest relatives of this particular group of apameine genera will be found elsewhere. Given the unique occurrence of the single genus of bamboo native to North America, and with most of the closest relatives of *Arundinaria* confined to temperate and subtemperate montane regions of the eastern Palearctic and Oriental Regions (Judziewicz et al. 1999), we suggest that the closest relatives of these apameine moth genera will be found there as well. Whether these seemingly unique morphological features are shared with as yet undiscovered species in Asia, and whether they are synapomorphic or symplesiomorphic relative to the rest of the Apameini remains to be determined. Such speculation might seem inappropriate, were it not provided with the hope that workers in the eastern Palearctic and Oriental Regions might pay special attention to any of the subtribe Arundinariinae Bentham, and any evidence of either endophagous or external feeding by noctuid larvae upon the culms of the various monopodial bamboo species occurring there. The montane regions of southern China west at least as far as Assam would appear particularly inviting. It is interesting to note that two of the three species of the genus *Enodia* Hübner (Lepidoptera: Nymphalidae: Satyrinae: Satryini) occurring in the United States are also known specialists upon *Arundinaria*, and that their closest relatives within the Satyrini (Wahlberg 2014) occur in southeastern Asia (e.g., Leech 1892-93). It should be pointed out that, aside from their sedentary nature, many of the apameine moths associated with *Arundinaria* in North America do not respond well to UV lights or traps, even when such are deployed directly within the canebrakes where the adult moths are actively breeding. Quinter has repeatedly observed mating and oviposition behavior of numerous individuals by searching these habitats with a flashlight at night, yet traps placed in or near these same sites yielded few or no specimens the following day. By far the most productive method for detecting the presence of some of these species is daytime searching for evidence of larval feeding, whether endophagous or external, and nocturnal searching for externally feeding climbing cutworms which rest in detritus lodged on the culms of the host plants, or on the forest floor, by day. For these reasons, almost all of these recently discovered species were previously unknown.

## Materials and methods

Photographic methods used herein are described in Sullivan and Adams (2009). Procedures for dissecting and preparing genitalia follow that of Lafontaine (2004). Terminology for genital structures and wing markings follows that of Lafontaine (1987, 2004). DNA sequencing of the barcode fragment of the CO1 gene was carried out at the Canadian Center for DNA Barcoding in Guelph, Ontario. Barcode sequences were compared by nearest neighbor analyses as implemented on the Barcode of Life Data systems website (Ratnasingham and Hebert 2007).

### Repository abbreviations

BMNH The Natural History Museum, London, UK

ELQ Eric L. Quinter, Willimantic, Connecticut, USA

CNC Canadian National Collection of Insects, Arachnids and Nematodes, Ottawa, Canada

JBS J. Bolling Sullivan, Beaufort, North Carolina, USA

USNM National Museum of Natural History, Washington, District of Columbia, USA

## Systematics

### Cherokeea

Taxon classificationAnimaliaLepidopteraNoctuidae

Quinter & Sullivan gen. n.

http://zoobank.org/DF48154A-49CD-4C62-A82A-3490032DD253

#### Gender.

Masculine.

#### Type species.

*Cherokeea attakullakulla* Sullivan & Quinter, 2014

#### Etymology.

*Cherokeea* is derived from Cherokee, a Nation of Native American people who occupied the southern Appalachians and were exemplary stewards of the habitats and resources of the region.

#### Diagnosis.

This genus exhibits most but not all of the primary characteristics of the tribe Apameini, i.e., ovipositor heavily sclerotized and dorsoventrally flattened, rugose sclerotized appendix bursae, and medially corrugated ductus bursae in the female; pleural sclerite a double helix in the male. It is distinguished from all known Nearctic and western Palearctic apameine genera by the conspicuous asymmetry of the saccular lobes of the male genitalia. This condition appears to be a uniquely derived synapomorphy shared with other as yet undescribed apameine genera restricted to southeastern United States. A sclerotized medial protrusion arising caudally from the basal margin of the male juxta appears to be autapomorphic. Additionally, the left valve bears a minute setose projection at the base of the sacculus, resembling a miniature clavus, which may be autapomorphic. The sole included species, *Cherokeea attakullakulla*, is a small, dull-colored moth bearing a superficial resemblance to some species of *Neoligia*.

*Cherokeea* is immediately distinguishable from *Neoligia*, *Oligia* Hübner, *Mesoligia* Boursin, and *Mesapamea* Heinicke by quite different genitalic morphology given in the description below. Troubridge and Lafontaine (2002) characterized the genera related to *Neoligia*. *Oligia* is differentiated by an elongated pollex (spatulate and setose) that projects from the base of the cucullus, a prominent digitus, a uniquely bent uncus and unarmed vesica in the aedeagus. *Mesapamea* has a paddle-like cucullus, no pollex, and an embedded digitus; the vesica has a basal cornutus. *Mesoligia* combines characters of the first two genera by lacking a distinct pollex, the digitus is embedded but more sclerotized, the uncus is similar to that of *Oligia* and the vesica contains a field of cornuti near the apex. *Neoligia* has a smooth pollex, a plate-like digitus fused to the inner surface of the valve but not projecting over the anal edge, and the vesica has both a basal cornutus and an apical field of small cornuti. None of these characters aptly fits the rarely-collected apameine of the southern Appalachians, described herein.

#### Description.

**Head.** Male and female antennae simple, setose-ciliate; 54 segments. Eye smooth, round. Labial palpus of both sexes laterally flattened, upcurved; first segment swollen basally, arching slightly upward and somewhat more than half as long as second segment, which is straight; second segment about as long as eye width, broadly scaled; third segment 1/3 × length of second, narrowly scaled, and projecting slightly anteriorly. Frons convex, unmodified; with a central dense tuft of converging spatulate hairs. **Thorax.** Vestiture a mixture of coarse spatulate scales, spatulate hairs and simple hairs; mesoscutellar crest prominent, metascutellar tuft, less so. **Wings.** Forewings elongated and acutely rounded at apex. Venation typical apameine, as figured in Mikkola et al. (2009) except that R3 and R4 are stalked for half the distance from the areole to the margin. **Legs.** Normal apameine; tibia devoid of spiniform setae, but with the usual pair of spurs on the mesothoracic leg and two pairs on the metathoracic leg. Epiphysis on prothoracic leg 0.5 × length of tibia; prothoracic tibia 1.3 × length of first tarsomere. Tarsus with three rows of spiniform setae on first two proximal tarsomeres; four irregular rows on distal three tarsomeres. **Abdomen.** First segment lacking paired, lateral coremata; eighth sternite with deciduous, non-eversible brush. A prominent mid-dorsal tuft on A1; no tufts on remaining segments. **Male genitalia** (Figs 5, 6). Uncus long, slender and downcurved to sharp apex; fine, long setae on outer half of dorsal surface. Tegumen broad at base of uncus, then flaring laterally, forming broad peniculi before narrowing sharply at pleural sclerite to meet U-shaped vinculum; distal edges of peniculi covered with fine setae. Saccus short, blunt, broadly V-shaped. Juxta an elongated trapezoidal shield, 2 x as long as basal width; a medial protrusion with a keyhole-like center extending caudally from the basal margin. Anellar arms not fused. Valve with subapical “neck” defining cucullus; ventral margin of valve slightly convex to ¾ from base, then abruptly angled dorsally into deep notch at anteroventral edge of cucullus; dorsal margin of valve evenly concave to ¾ from base, then abruptly angled dorsally to form rounded process at anterodorsal edge of cucullus. Cucullus triangular, apically slightly spatulate, with corona reduced to one or two apical setae; outer margin of cucullus bearing several larger spines, including two prominent anal spines; inner face of cucullus with a patch of fine hairs, denser apically. Valves bilaterally asymmetrical with respect to shape of sacculus. Left valve bearing a minute setose projection at base of sacculus, resembling a miniature clavus, which appears autapomorphic; otherwise, costal lobe of left sacculus normal, rounded; costal lobe of right sacculus greatly expanded dorsally into a free, flattened process that extends distally half length of entire valve; saccular lobe attached at its distal base to more heavily sclerotized basal sclerite of clasper. Basal sclerite of clasper a narrow, sclerotized bar subparallel to ventral margin of valve, terminating in a point that fuses indistinguishably with digitus. A slender, setose ampulla projects posterodorsally from dorsal arm of clasper; ampulla 7–10 × as long as wide. Costal margin of valve heavily sclerotized, becoming free from surface of valve toward cucullus to form digitus. Digitus abruptly angled near neck of cucullus to project posteroventrally along anteroventral margin of cucullus, dorsal arm shorter, projecting in opposition; dorsal arm fused with rounded process at anterodorsal edge of cucullus; free, curved, ventral arm extended length of cucullus. Aedeagus (1.6 mm; n = 7, 1.5–1.8 mm) curved ventrally, 5–6 × as long as wide, with sclerotized band extending onto basal 1/4 of vesica on left. Vesica (3.5 mm; n = 6, 3.4–3.7 mm) kidney shaped, about 2 × as long as aedeagus, curving to right through 180° to project anteriorly; without basal or subbasal cornuti, but with two basal sclerotized straps projecting on to base of vesica, and a single spine-like bundle of smaller subparallel spines projecting distally near apex of vesica.

**Female genitalia** (Fig. 7). Posterior tip of papillae anales to anterior end of corpus bursae 7.2 mm; n = 2, 6.6–7.8 mm. Corpus bursae membranous, elongate, 2 × as long as wide, ovoid, slightly constricted posterior to middle, without signa. Appendix bursae corrugated, arising posteriorly on left, more heavily sclerotized distally, 0.8 × length corpus bursae. Ductus bursae long, narrow, 12 × as long as wide, 0.8 x length corpus bursae, heavily sclerotized in longitudinal ridges, wider anteriorly than posteriorly, entering at their junction on right side 1/3 distance from posterior end of appendix bursae to anterior end corpus bursae. Lamella antevaginalis quadrate with W-shaped outline, sclerotized, strongly indented anteriorly at juncture with ostium, somewhat concave posteriorly; dorsal wall of ostium membranous, lacking any discernible lamella. Anterior and posterior apophyses 1.5 × length A8, slender with paddle-like terminations. Papillae anales dorsoventrally flattened, evenly tapered, acutely pointed cones with dorsal surface densely spinulose, ventral surface minutely setose. The two sclerotized rods between the anal papillae characteristic of Apameini apparently secondarily lost in the very small adults.

### Cherokeea attakullakulla

Taxon classificationAnimaliaLepidopteraNoctuidae

Sullivan & Quinter sp. n.

http://zoobank.org/F8CF625C-480D-4BE2-9354-13CAEA2C388A

Figs 1 –7

#### Type locality.

Fontana View Estates on Lake Fontana, Swain County, North Carolina.

#### Type material.

**Holotype male:** USA, North Carolina: Swain County, 2000’, Fontana Lake Estates (35°38.44'N, 83°55.79'W), mesic mixed pine/hardwoods June 10, 2002. J. Bolling Sullivan (USNM). **Paratypes:** (9♂ 12♀) same data as holotype, 8 and 10 June, 2002 (BMNH, USNM, CNC, ELQ, JBS). **Other material examined:** over 100 of both sexes collected from June 8–24 from: Great Smoky Mountains National Park, Swain County, North Carolina (Wiggins-Watson Cemetery, Deep Creek, 2215’ (35°28.0'N; 8326.2'W); Forney Creek, 1840’ (35°28.1'N, 83°34.0'W.); Big Cove Road, 2054’ (35°51.6'N, 83°29.4'W); Welch Ridge, 1840’ (35°26.9'N, 83°44.6'W); Rutherford County, North Carolina, Box Creek Preserve, 1100–1500’ (35°54.8'N; 81°93.9'W).

#### Etymology.

The name Attakullakulla, herein treated as a noun in apposition, refers to the Supreme Cherokee Leader (from 1761–1775) who represented his people in London in 1730 and at home in the Carolinas while negotiating various peace treaties.

#### Diagnosis.

The very limited distribution of this species to moderate altitudes in the southern Appalachian Mountains and foothills is unlikely to coincide with any species of *Neoligia*. It is possible the moth might be confused with worn specimens of the common, widely distributed eustrotiine moth *Protodeltote muscosula* (Guenée), but adults of that species are slightly larger, with an olivaceous cast to the forewings, a prominent, subquadrate dark patch between the orbicular and reniform spots, and lack the characteristic genitalic features of Apameini. Otherwise, the genitalic characters described under the genus will serve to distinguish this species from anything of similar appearance occurring in North America.

#### Description.

**Figures 1–4.**
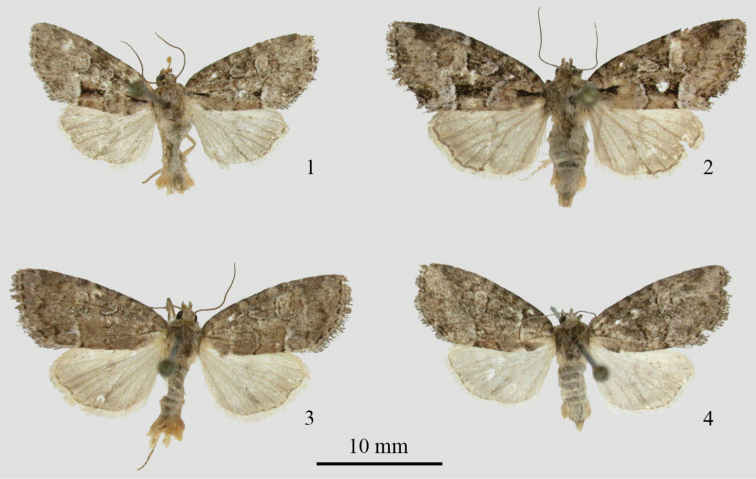
*Cherokeea attakullakulla* adults. **1** male holotype **2** female paratype, mottled form **3** male paratype, plain form **4** female paratype, plain form.

**Head.** Dorsum of antenna with alternating brown and gray rings of scales, underside tan; scape white. Labial palpus with gray and brown rough scaling. Frons with a mixture of white and brown erect scales, vertex and collar similar but with more dominant brown scaling. **Thorax.** Vestiture a mixture of coarse, brownish, spatulate scales, spatulate hairs and simple hairs; mesoscutellar crest prominent, metascutellar tuft, less so. **Legs.** As detailed in generic description. **Wings** (Figs 1–4). Forewings elongated and acutely rounded at apex. Forewing base to wing tip, 10.7 mm; N=20, (9.8–11.7 mm). Appearance variable, ranging from nearly uniform dull gray to much more contrasting and mottled, especially in females. Both phenotypes occur in both sexes, however. Antemedial line excurved, doubled, with pale gray to nearly white filling; slightly scalloped and comprised of black scales. Medial line or shade obscure. Postmedial line sinuous, excurved around reniform, most distinct at posterior margin, becoming obscure toward costa; slightly scalloped and doubled, with pale gray to nearly white filling; inner element of pm line much darker than the outer, which is defined by black points on veins. Subterminal line a merged series of brownish-black indistinct chevrons. Terminal line a series of sharp, tiny black chevrons between veins. Fringe pale gray, with unbroken dark gray basal line. Basal, medial and terminal areas predominantly uniform gray, but with some reddish-brown scaling in the medial and basal areas in some individuals, especially toward posterior margin. Subterminal area usually paler gray, with quadrate, subapical dark patch on costa. Basal dash usually clearly visible, black, often highlighted below with whitish scales; medial dash variable, from completely obscure to a prominent bar, which may be the most distinct marking of the forewing; anal dash obscure or, at most, represented by an indistinct, dark, triangular patch of scales. Reniform spot auriculate, pale gray, of same shade as subterminal area. Orbicular spot similarly colored, ovoid, with an outline of black scales. Suborbicular and claviform spots not visible on worn material studied. Dorsal hindwing pale gray, plain, unmarked except for faint discal spot; fringe pale gray with darker gray basal line. **Abdomen.** Dorsal abdominal scaling white basally then brownish to tip; a prominent mid-dorsal tuft on first segment; ventral abdominal scaling brownish, becoming more yellow on ventral brush on eighth segment. **Genitalia.** As detailed for both sexes in generic description.

**Figures 5–7.**
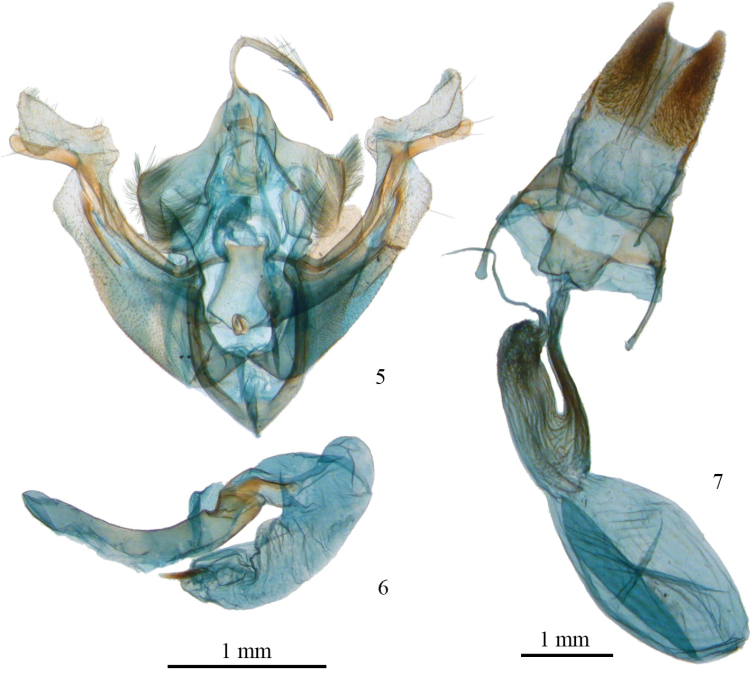
Genitalia structure of *Cherokeea attakullakulla*. **5** male genitalia (aedeagus removed) (JBS5761) **6** male aedeagus and vesica (JBS5761) **7** female genitalia (JBS5757).

#### Molecular results.

Barcodes were obtained for seven specimens from both Swain and Rutherford Counties. There were five haplotypes which differed by as much as 0.6%. The most common haplotype was:

AACATTATATTTTATTTTTGGAATTTGAGCAGGTATAGTTGGAACCTCTTTAAGATTACTAATTCGAGCTGAATTAGGAAACCCCGGATCTTTAATTGGTGACGATCAAATTTATAATACTATTGTTACAGCTCATGCTTTTATTATAATTTTTTTTATAGTTATACCTATTATAATTGGAGGATTTGGAAATTGACTTGTACCTTTAATATTAGGAGCTCCAGATATAGCATTTCCACGAATAAATAATATAAGTTTTTGGTTACTTCCCCCATCTTTAACTTTATTAATTTCAAGAAGAATTGTAGAAAATGGAGCTGGAACAGGATGAACAGTGTACCCCCCACTTTCATCTAATATTGCTCACGGAGGAAGTTCTGTAGATTTAGCCATTTTTTCTCTTCATTTAGCTGGTATTTCTTCTATTTTAGGAGCTATTAATTTTATTACTACAATTATTAATATACGATTAAATAATTTATCTTTTGATCAAATACCTTTATTTATTTGAGCGGTAGGAATTACTGCATTTTTATTATTATTATCACTACCCGTTTTAGCGGGAGCTATCACAATATTATTAACAGATCGAAATTTAAATACATCTTTTTTTGATCCTGCAGGAGGAGGAGATCCAATTTTATATCAACATTTATTT

Barcode sequences for *Cherokeea attakullakulla* did not associate with *Neoligia* or other related genera when nearest neighbor similarity searches were conducted.

Two patterns of maculation are seen (Figs 1–4), which we are calling mottled and plain. Intermediates are seen as well. These phenotypes did not segregate by location or by barcodes. Females are usually more heavily marked than males.

### Immature stages

We have not yet had the opportunity to investigate the bionomics of this species fully, so the early stages remain unknown. Based upon habitat association, unequivocal placement within the Apameini, and especially details of genitalic morphology clearly linking this species to other taxa known to be specialists upon *Arundinaria*, it appears virtually certain that its larva will be found to be an endophagous feeder upon *Arundinaria appalachiana* Triplett, Weakley & L.G. Clark (Triplett et al. 2006), the native grass occurring at the locations and altitudes where the adult moths were collected.

### Distribution and habitat

In North Carolina the species is known from Swain and Macon Counties in the mountains and from Rutherford County in the foothills. The only record for Georgia is Rabun Co., slopes of Rabun Bald, 0.7 road mi. past Kelsey Mtn. Road parking lot, 4000’, June 21, 2001, James Adams (Adams, personal communication). The species is univoltine and flies from the 8^th^ through the 24^th^ of June. It is always found in association with hill cane (*Arundinaria appalachiana*), which grows on well-drained forested slopes. Two other species of *Arundinaria* occur in North Carolina but are associated with wet habitats, are often over 6’ in height, and grow in dense colonies. Hill cane grows singularly or in poorly defined clumps and is less than four feet in height and can be found on rocky knobs, hillsides, and throughout mesic oak-hickory forests in the foothills and lower mountains (up to about 3000’).

## Discussion

Despite well-developed flight musculature, most adult apameine moths are highly sedentary and non-vagile. As a result, many of the species occur only in small, extremely localized populations and thus are infrequently collected, creating a false impression of rarity, when in fact they can be extremely abundant within their respective niches. Hence the availability of suitably fresh study material for molecular analysis represents a major challenge. Some of the earlier molecular analyses were based on a very limited subset of the species occurring in a region, and of relatively little value as a consequence. More recent work in North America and Europe has a high degree of species-level coverage in the 94% range (J.D. Lafontaine, pers. com.). Issues of generic non-monophyly are thus revealed. Phylogenetic studies based on morphology have one advantage in that comprehensive representation is more readily achievable.

Morphological studies of Lepidoptera in general and Noctuoidea in particular (e.g., Forbes 1948, 1954, 1960) traditionally have relied heavily upon genitalic structure, especially of adult males. Female genitalic characters were too often ignored. What has become most apparent in the broad overview of such studies is that the genitalic characters so emphasized range from practically useless even as diagnostic for species-level taxa, much less indicators of phylogenetic relationships among them, to moderately useful in other instances, to extremely so in certain groups, notably the Apameini. This in large part likely reflects the various evolutionary mechanisms operating in different groups. In those lepidopteran groups where diurnal behavior or temporal isolation mechanisms function, or where pheromone-driven selection mechanisms prevail, there may be little need for adaptive modifications of the genitalia. Where genitalia have evolved into so-called “lock-and-key” type of function, as apparent in the Apameini (Mikkola 1992), genitalic character states may be extraordinarily promising indicators of phylogenetic relationships. The Apameini have evolved extremely complex genitalic morphology, the most complex within the Noctuoidea, and perhaps even of all Lepidoptera. The long history of debate over whether these morphological adaptations truly function as reproductive isolation mechanisms has been revisited recently and summarized Masly (2012). Many such claims have been rejected in the past as untestable, but Masly suggests the future may be brighter in this regard. Mikkola (1992) left little doubt that apameines remain ideal subjects for such study. These moths are clearly well suited for phylogenetic analyses involving combined morphological and molecular data. The inclusion of data regarding the immature stages will render such analyses even more robust. The bionomics of western Palearctic taxa are relatively better known than those of North America, but much progress is being made here as well.

Even with regard solely to morphology, global treatments have thus far been quite uneven. The apameine fauna of the western Palearctic recently have been comprehensively studied and documented (Zilli et al. 2005). That work now serves as a model of what is needed elsewhere. More recent work (Mikkola et al. 2009) has made significant progress toward elucidating certain Nearctic apameine taxa and unifying the nomenclature across the Holarctic Region. Much work remains to be done in North America, however. The present paper represents one small step in that direction, with related studies in progress. It is hoped that a global review incorporating molecular data will ultimately result in a clearer understanding of the phylogenetic relationships of these fascinating insects.

## Supplementary Material

XML Treatment for Cherokeea

XML Treatment for Cherokeea attakullakulla
